# The extremely rare vascular variant of a segmental duplicated uterine artery and its relevance for the interventionist and gynecologist: a case report

**DOI:** 10.1186/s13256-016-0943-2

**Published:** 2016-06-04

**Authors:** Gernot Rott, Frieder Boecker

**Affiliations:** Department of Radiology, Bethesda-Hospital Duisburg, Heerstrasse 219, 47053 Duisburg, Germany

**Keywords:** Uterine artery, Uterine artery embolization, Uterine fibroid embolization, Anatomic variant, Vascular anomaly, Duplicated artery

## Abstract

**Background:**

Anatomic variants of the uterine artery are rare, with the absence of one of the uterine arteries presumably being the most abundant variant. A duplicated uterine artery is mentioned in the medical literature, but to the best of our knowledge, an angiographic study has never been published. A partially duplicated uterine artery is an extremely rare variant not previously mentioned in the literature, and it could lead to technical difficulties or cause problems in various gynecological interventions.

**Case presentation:**

We present the case of a 45-year-old Caucasian woman with a uterine fibroid and typical fibroid-related symptoms who came to our department to get treated with fibroid embolization. During the procedure, angiography revealed a partial or segmental duplicated left uterine artery. This exceptionally rare anatomic variant proved to be beneficial for the safety of the embolization in our case; however, it is far more likely that such a variant would be unfavorable in some types of gynecological operative and minimally invasive techniques.

**Conclusions:**

Knowledge of the anatomic variant of a partially duplicated uterine artery is important, especially for gynecologists performing minimally invasive surgical procedures.

## Background

Uterine fibroid embolization (UFE) is a common interventional radiological procedure with high technical success and effectiveness in experienced hands. Anatomic variants of the uterine artery can present challenging during UFE and mainly concern the origin of the vessel. We describe a previously unpublished variant of a partially duplicated uterine artery and discuss its clinical significance for interventional radiologists and gynecological surgeons.

## Case presentation

A 45-year-old Caucasian woman with typical bleeding and pressure symptoms caused by a solitary intramural fibroid contacted our Interventional Radiological Department, asking whether she could be offered UFE. A gynecological examination with ultrasound verified the diagnosis of a fibroid on the back wall of her uterus measuring about 7 cm and confirmed the indication for UFE.

**Fig. 1:**
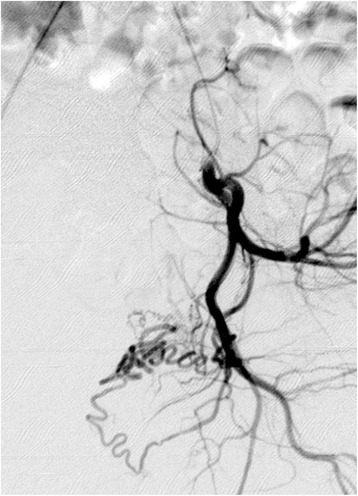
Left internal iliac arteriogram showing the left uterine artery in a seemingly normal form and shape

**Fig. 2:**
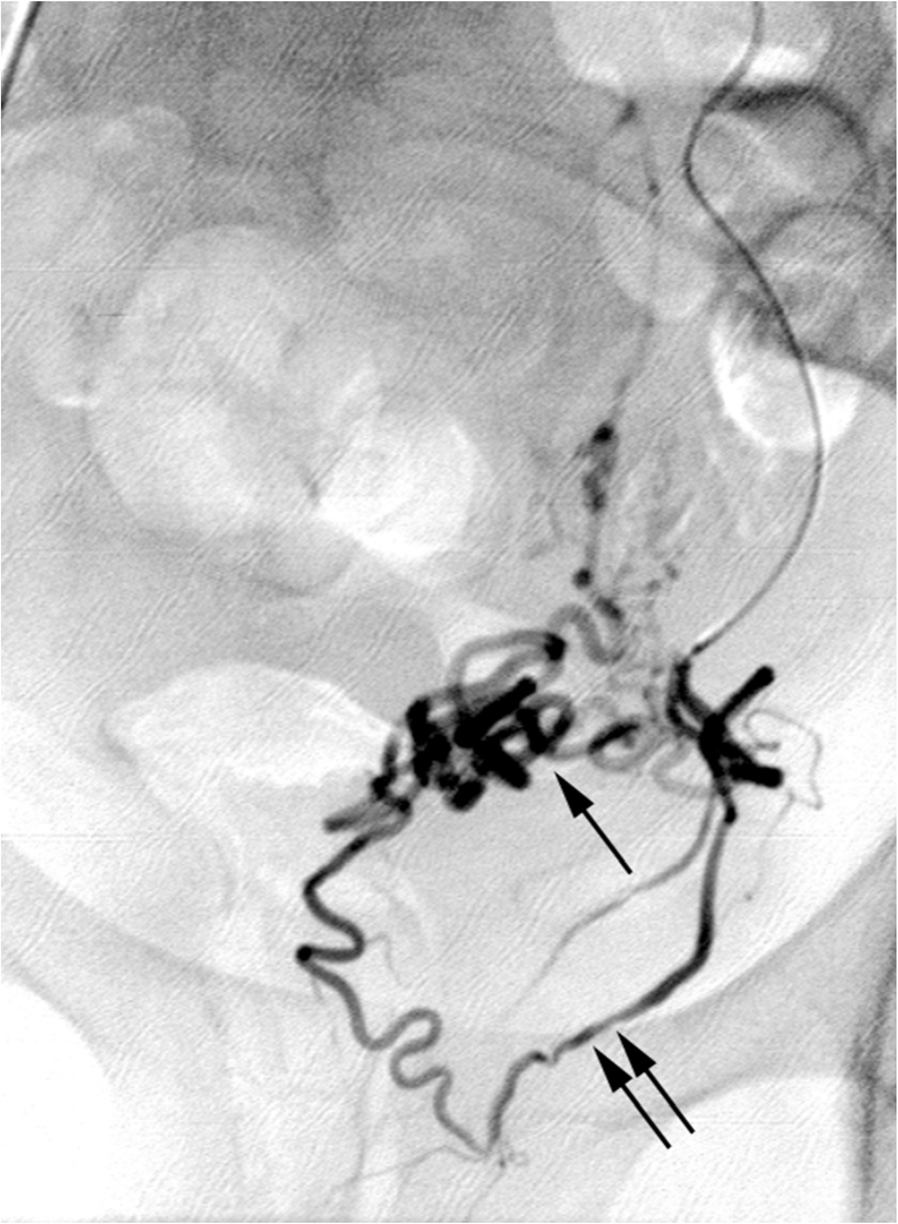
Selective angiography of the left uterine artery via a microcatheter shows the distribution of the uterine artery into two transverse segments (U2 segments): an upper segmental (arrow) and a lower segmental (double arrow) branch

**Fig. 3:**
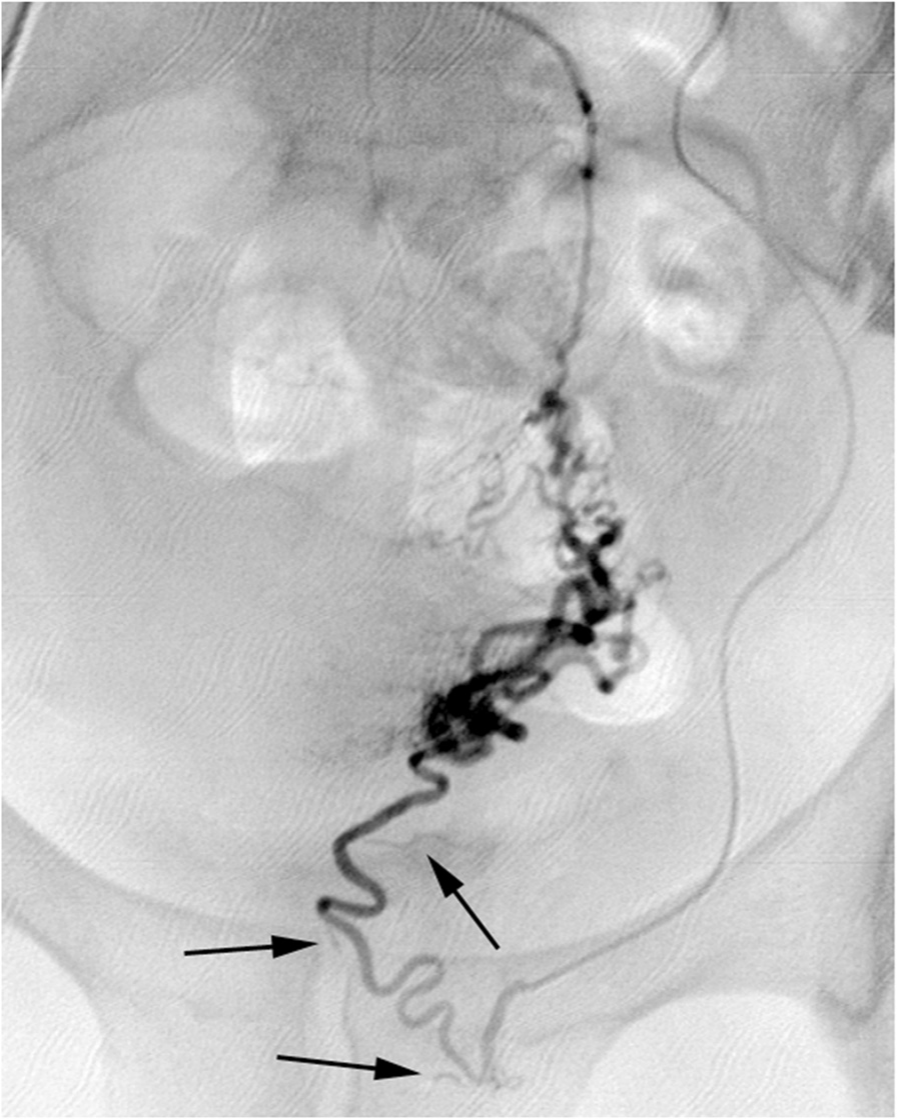
Superselective angiogram of the lower transverse segment (lower U2 segment) of the uterine artery with small vaginal branches (arrows)

**Fig. 4:**
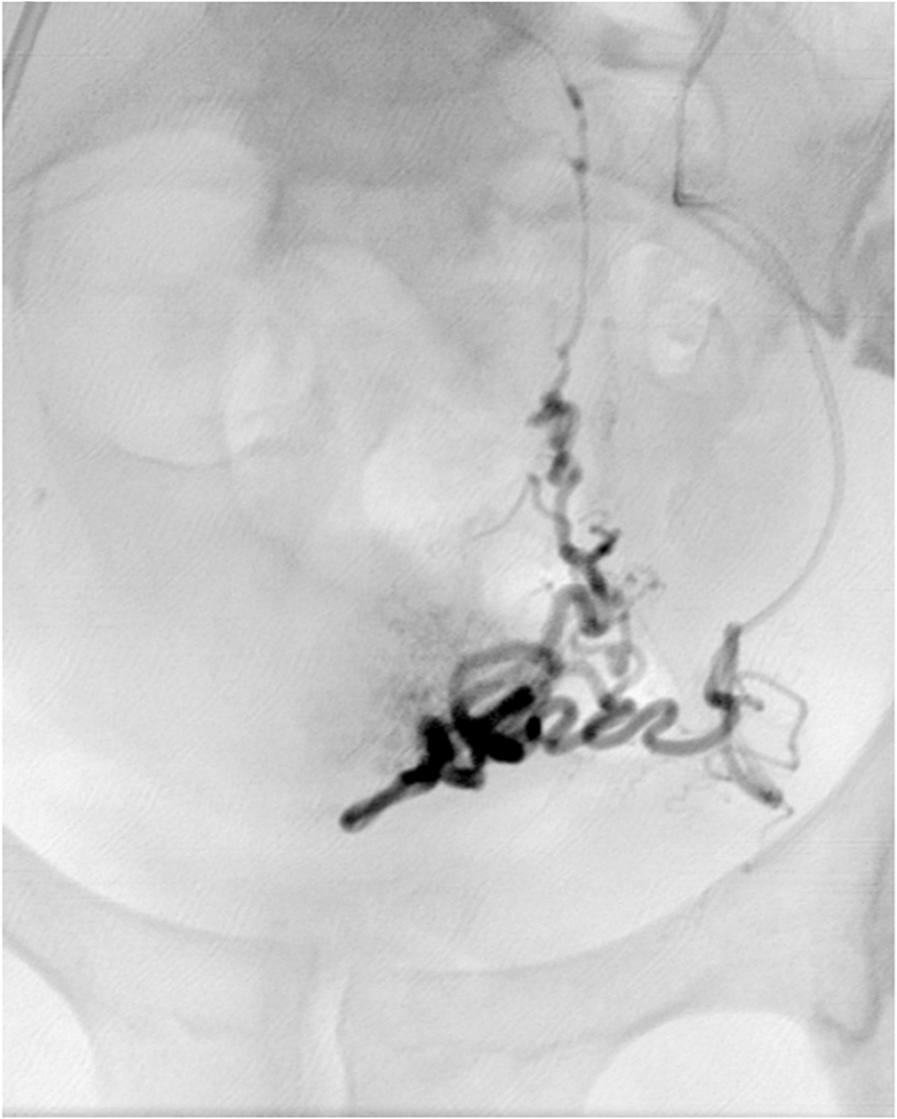
Superselective angiogram of the upper transverse segment (upper U2 segment) of the uterine artery

After standard preparation, arterial access was obtained by the retrograde puncture of her right femoral artery and insertion of a 5-Fr sheath (Radifocus Introducer II, Terumo Corporation, Tokyo, Japan). Angiography of the left internal iliac artery was performed in a crossover maneuver with a 4-Fr diagnostic catheter (Cobra II, Terumo Corporation). This revealed that her left uterine artery was relatively small but exceptionally twisted and tortuous (Fig. 1). Selective arteriography using a microcatheter (2.7-Fr; Progreat, Terumo Corporation) revealed a bifurcation of her left uterine artery prior to the junction of the descending and transverse portions, with one branch crossing several centimeters above the other before rejoining at the ascending portion (Fig. 2). Superselective catheterization of both branches of the transverse portion of the uterine artery confirmed the unusual anatomic variant (Figs. 3 and 4). In particular, the lower transverse branch showed all the characteristic criteria for a uterine artery, in form and shape, supplying blood solely to the uterus. Because only the lower segment of the transverse portion had additional small vaginal branches, the upper segment was chosen for embolization of her left uterine artery.

The embolization was subsequently performed using calibrated microspheres measuring 500–700 μm (Embosphere Microspheres, Biosphere Medical, Paris, France). Access to her right uterine artery was also obtained using a coaxial technique and a 5-Fr diagnostic catheter (RUC, Cook Medical, Bloomington, IN, USA) and the above-mentioned microcatheter. Embolization of her right uterine artery was performed in the appropriate manner. Angiography showed that the fibroid had a strongly right dominant vascular supply.

Our patient’s post-intervention course was uneventful and she was discharged 2 days later. In the follow-up questionnaire 3 months later, our patient expressed herself sufficiently satisfied with the result of the UFE and stated that she would choose the procedure again.

## Discussion

In anatomic terms, the uterine artery runs down the retroperitoneal space of the lateral pelvic wall (first or descending segment), travels intraperitoneally through the broad ligament (second or transverse segment), and then gives off ascending branches to the uterine body and descending branches to the cervix uteri at the uterine margin (third or ascending segment).

On angiography, the artery has an overall u-shaped appearance, and the transverse and ascending segment are typically markedly tortuous to ensure a sufficiently large reserve capacity for vessel elongation during pregnancy.

In terms of embryology, the uterine artery develops as a branch of the anterior division of the internal iliac artery, which in itself develops from the patent part of the umbilical artery. The origin of the uterine artery in human fetuses in most cases arises separately from the internal iliac artery [1]. The three segments of the uterine artery, the descending, transverse, and ascending segment, hereafter referred to as the U1, U2, and U3 segment, are clearly visible in the fetus by no later than 5 months [2].

Anatomic variations of the uterine artery are frequently found in its origin and side branches, the latter of which may be named collaterals or anastomosis. The uterine artery can be classified into four types depending on its origin. Type I originates as the first branch, and type II as the second or third branch of the anterior division of the internal iliac artery. Type III originates as a trifurcation of the anterior and posterior divisions of the internal iliac artery. Type IV originates as the first branch of the internal iliac artery [3].

Side branches of the uterine artery mainly supply the vagina (vaginal branches), the ovaries (ovarian branches), the Fallopian tubes (tubal branches), or the uterus itself (via intramural arcuate vessels, also known as helicine branches). Excepting the latter, they have variable development, and in this respect are visible on angiography with varying frequency [4].

Other anatomic variations of the uterine artery are rare. The uterine artery may have a common trunk with another branch of the internal iliac artery, for example, with the vesical, the vaginal, or the internal pudendal arteries [4, 5]. Saraiya *et al*. reported the replacement of the uterine artery by the round ligament artery [5]. Pelage *et al*. stated that the uterine artery may be replaced by multiple small artery branches and presented a flush pelvic aortogram as an example of this [4]. The same observation, with only multiple small branches lacking a distinct uterine artery trunk on either side, was made during a uterine artery embolization by Worthington-Kirsch *et al*. [6], who for that reason had to abandon the procedure.

The supposed existence of the duplication of the uterine artery has been reported by several authors; however, in most cases this has only been in the form of secondary literature [4, 7]. In researching the primary literature, we were not able to find a single case of a duplicated uterine artery proven on angiography, because, as mentioned above, variants seem to occur as the absence of a uterine main trunk with replacement of the uterine artery by multiple small vessels originating from the internal iliac artery supplying the uterus [6]. We only came across one reported case of a duplicated uterine artery, discovered and photographed during a laparoscopic hysterectomy [8].

Our presented case of a duplication of the U2 segment of the uterine artery is clearly and angiographically proven to be a variant of a partially duplicated uterine artery. It was not a replacement of the uterine artery by several small anonymous vessel branches, nor a uterine artery with an extraordinary large vaginal branch, nor a uterine artery sharing a common trunk with the vaginal or any other artery.

Anatomic variants of arteries may interfere with the safety and success of any transcatheter embolization procedure. The clinical relevance of the described variant of a partial duplicated uterine artery for the interventional radiologist is exemplarily illustrated by our case. If it is possible to advance a catheter in both of the U2 segmental branches, the more suitable one can be chosen for embolization of fibroids to minimize or avoid the risk of misembolization of the vagina. Because both segmental branches of a duplicated uterine artery will present with rather small vessel calibers, this manoeuver does, however, risk uterine artery vasospasm, possibly leading to undertreatment or treatment failure in fibroid embolization. If it is not possible to advance the catheter superselectively into one of the U2 segmental branches, the embolization has to be performed from the U1 segment, accepting the risk of potential misembolization of vaginal branches and very rare but described complications such as sexual dysfunction [9].

For the gynecological surgeon, unexpected anatomic variations during surgical procedures may lead to challenging clinical scenarios. Knowledge of anatomical variants, particularly concerning the arterial supply of targeted organs, is mandatory for surgeons to prevent unintended vessel injury and potentially serious complications. This applies especially to minimally invasive laparoscopic operations. During these interventions, several methods exist to gain access to the uterine vessels, irrespective of whether a hysterectomy, myomectomy, or another procedure is performed. The uterine artery is usually ligated, coagulated, or clipped near the neck of the uterus at the U3 segment or at its origin from the internal iliac artery (U1 segment), but also at the U2 segment within the broad ligament [10, 11]. For many surgical procedures, the intervention begins with a retroperitoneal dissection or transection of the broad ligament to identify the uterine artery, its course, and the ureter [12–14]. However, unlike angiography, using laparoscopic approaches to access the uterine vessels cannot necessarily rule out vascular anomalies. After ligation or clipping of the one and presumably only uterine artery on the corresponding side, the gynecologist does not expect another artery during this step of surgical preparation. To then come across another completely unexpected artery, a second U2 segment of the uterine artery, may cause vessel injury with excessive arterial bleeding and consecutive restricted visibility of the surgical field, and result in the need for conversion to open surgery and/or a blood transfusion.

Technical difficulties in approaching and skeletonizing the uterine vessels occur particularly in the case of large myomas or large uteri. At least in these cases and in the context of uterine artery occlusion procedures, such as transvaginal uterine artery ligation or temporary transvaginal uterine artery clamping [15, 16], a uterine artery with a duplicated U2 segment could present a very unpleasant surprise and lead to technical difficulties, treatment failures, or even severe complications.

## Conclusions

A partial or segmental duplicated uterine artery with a duplication of the transverse segment (U2 segment) is an extremely rare anatomic variant. Knowledge of this variant is important, especially for the gynecologist performing minimally invasive surgical procedures.

## Abbreviations

UFE, Uterine fibroid embolization
